# Social Context and Reward Sensitivity Enhance Corticostriatal Function during Experiences of Shared Rewards

**DOI:** 10.1101/2023.10.19.562908

**Published:** 2023-10-19

**Authors:** Ori Zaff, James B. Wyngaarden, Jeffrey B. Dennison, Daniel Sazhin, Jason Chein, Michael McCloskey, Lauren B. Alloy, Johanna M. Jarcho, David V. Smith, Dominic S. Fareri

**Affiliations:** 1 Department of Psychology & Neuroscience, Temple University, Philadelphia, PA, USA; 2 Derner School of Psychology, Adelphi University, Garden City, NY, USA

## Abstract

Although prior research has demonstrated enhanced striatal response when sharing rewards with close social connections, less is known about how individual differences affect ventral striatal (VS) activation and connectivity when experiencing rewards within social contexts. Given that self-reported reward sensitivity and level of substance use have been associated with differences in VS activation, we set out to investigate whether these factors would be independently associated with enhancements to neural reward responses within social contexts. In this pre-registered study, participants (N=45) underwent fMRI while playing a card guessing game in which correct or incorrect guesses resulted in monetary gains and losses that were shared evenly with either a close friend, stranger (confederate), or non-human partner. Consistent with our prior work, we found increased VS activation when sharing rewards with a socially close peer as opposed to an out-of-network stranger. As self-reported reward sensitivity increased, the difference in VS response to rewards shared with friends and strangers decreased. We also found enhanced connectivity between the VS and temporoparietal junction when sharing rewards with close friends as opposed to strangers. Finally, exploratory analyses revealed that as reward sensitivity and sub-clinical substance use increase, the difference in VS connectivity with the right fusiform face area increases as a function of social context. These findings demonstrate that responsivity to the context of close friends may be tied to individual reward sensitivity or sub-clinical substance use habits; together these factors may inform predictions of risk for future mental health disorders.

## Introduction

From purchasing a car to getting engaged, important life decisions are often made in social contexts, whether around strangers or loved ones. Social influence can dramatically shape attitudes towards reward-related decision-making (Dennison et al., 2022; Fareri et al., 2012; Powers et al., 2022), including maladaptive choices (O’Brien et al., 2011). Neural reward valuation is also dependent upon individual differences in trait sensitivity to rewards (Chat et al., 2022; Scult et al., 2016). However, our understanding of how neural reward responses to social context varies based on individuals’ reward sensitivity is limited.

The ventral striatum (VS), a key region in reward processing (Haber & Knutson, 2010; Middleton & Strick, 2000), responds to both monetary and social rewards (Bhanji & Delgado, 2014). Rather than respond to social or monetary reward in isolation, the VS demonstrates an additive effect. For instance, sharing monetary rewards with friends (relative to strangers) has been associated with enhanced VS response (Fareri et al., 2012). Behavioral metrics of satisfaction, excitement, and social behavior across changing social contexts corroborate the change in VS response (Dziura et al., 2022; Fareri et al., 2012).

Social information is not processed in isolation. The temporoparietal junction is among regions implicated in a network involved in processing social information dubbed the “social brain.” (Bhanji & Delgado, 2014; Hutcherson et al., 2015; Lockwood et al., 2020; Mars et al., 2012a). The posterior TPJ is associated with social context processing, social attention, and mentalizing (Bukowski, 2018; Doricchi et al., 2022; Mars et al., 2012b; Santiesteban et al., 2017) and generosity is linked with increased VS-TPJ connectivity (Park et al., 2017; Tusche et al., 2016). Additionally, the fusiform face area (FFA) has been considered essential to the social brain’s processing of social context (Schultz et al., 2003) and demonstrates connectivity with the VS in response to shared rewards (Haeger et al., 2014). Connectivity between “social brain” regions and the VS may contribute to enhanced enjoyment when sharing rewards with friends. Moreover, direct evaluation of neural connectivity when sharing real monetary rewards could reveal how individual differences in reward processing alter responsivity to social context.

A key metric in understanding individual variation in the neural reward response is trait reward sensitivity, or the degree to which rewarding stimuli motivate approach behavior (Carver & White, 1994; Haber & Knutson, 2010; Kim et al., 2015; Nusslock et al., 2007). However, the relationship between reward sensitivity and social context in influencing the function of neural reward circuitry remains severely understudied (Sazhin et al., 2020). Aberrant approach motivation is linked to dysfunctional reward processing (Chat et al., 2022), mood disorders, and increased vulnerability to addiction and substance use (Nusslock & Alloy, 2017; Volkow et al., 2010), which are in turn linked to altered striatal connectivity (Kober et al., 2010). Furthermore, since both reward sensitivity and peer relationships may influence level of substance use (Franken & Muris, 2006; Shadur & Hussong, 2014; Strickland & Smith, 2014), accounting for interactions between an individual’s trait reward sensitivity and substance use may allow more accurate assessment of neural reward response in social contexts. Assessing how social context and trait reward sensitivity modulate corticostriatal function advances our understanding of psychopathologies characterized by maladaptive reward processing.

This study sought to investigate how trait reward sensitivity moderates the influence of social contexts on reward processing independent of sub-clinical substance use. We employed a card guessing task in which monetary outcomes were shared with different partners (computer, stranger, close friend) (Delgado et al., 2000; Fareri et al., 2012) while participants underwent functional magnetic resonance imaging (fMRI). Trait reward sensitivity and substance use level were examined as independent moderators of the neural response to rewards shared with different partners. In our pre-registered hypotheses, we predicted that the varied social contexts under which outcomes were shared would modulate behavioral and VS responses to shared rewards. We additionally predicted that neural activation and connectivity would be moderated by reward sensitivity and substance use independently.

## Materials and Methods

### Participants

Our initial pre-registered goal was to collect data from 100 participants (18–22). However, due to constraints imposed by the COVID-19 pandemic, we were ultimately able to collect data from 52 participants through at least one run of this task, as part of a broader experimental session. Using pre-registered exclusionary criteria (https://aspredicted.org/blind.php?x=SFX_MXL), some participants were excluded from analyses due to head-motion (N=3; i.e., both runs were motion outliers, characterized via quality measures from MRIQC (Esteban et al., 2017): fd_mean >1.5 times the upper bound of the interquartile range or tsnr values < 1.5 times the lower bound of the interquartile range). Other participants were excluded for failure to respond during the behavioral task (N=2; i.e., >20% missing responses), or incomplete data (N=2; failure to complete survey data or missing behavioral data due to technical issues). These exclusions resulted in a final sample of 45 participants (mean age: 21.11 years, SD: 1.83 years; 36.4% male). Each participant referred a same-gendered friend to our study. Friends were asked to submit a photo of their faces, which we used in the Shared Reward task described below.

Participants were recruited via the Temple University Psychology and Neuroscience Department participant pool, and from the surrounding community via flyers and online advertisements. Participants were paid $25 per hour for fMRI and $15 per hour for behavioral tasks, and received bonuses based on their decisions on other neuroeconomic tasks administered within the experimental session (not reported here), resulting in a $100 base payment and an average bonus of $50. Participants recruited from the University Pool received research credit hours for their participation in place of cash but were eligible for the monetary bonus payment based on their decisions.

### Procedure

Recruitment and procedural methods were approved by the Temple University IRB. Participants began the study by completing an initial interest screener. After a behavioral consent form, the screener involved completing the Behavioral Inhibition System and Behavioral Activation System Scale (BIS/BAS; Carver & White, 1994) and Sensitivity to Punishment and Reward Questionnaire (SPSRQ; Torrubia et al., 2001) on Qualtrics. Participants who provided similar responses on both measures of reward sensitivity (i.e., within one quintile) were contacted to participate in the study (Alloy et al., 2009).

Participants were additionally excluded if they were unwilling to abstain from drinking alcohol or using recreational substances within 24 hours of the MRI scan. Those taking psychoactive medications were not recruited. Participants who passed the screener were run through a mock version of the scan to train on reducing head-motion. A breathalyzer test and urine drug screen were then conducted to ensure that performance and/or brain activation at the time of the scan was not confounded by recent substance use or substances still detectable in participants’ systems. Of participants who passed the initial screener, two were excluded after testing positive for morphine and amphetamine usage. 5 participants who tested positive for marijuana were included in our final sample. Following these procedures, participants underwent fMRI for 1.5 hours, during which they spent approximately 15 minutes completing the Shared Reward task described below. After the scan, participants completed several additional tasks and surveys.

### Shared Reward Task

We administered a card-guessing game to participants undergoing fMRI (adapted from Delgado et al., 2000; Fareri et al., 2012) to assess responses to monetary rewards and losses experienced in social and non-social contexts. Participants guessed if the number on a card was higher or lower than 5. A question mark would appear on the screen, during which time participants had 2,500 ms to select ‘higher’ or ‘lower’ with their right index or pointer finger, respectively. Upon selection the mark would turn orange, and remain so until the phase was complete and an outcome was displayed. After this, the actual number on the card was displayed from a randomly generated parameter file, while an indication of win (green arrow pointing up), loss (red arrow pointing down), or neutral (i.e., card value equivalent to 5, white arrow pointing side-to-side) appeared above it. If participants failed to guess within the 2,500 ms during which the question mark was on the screen, a number sign was displayed in place of the actual number on the card indicating a missed trial. Correct guesses were associated with monetary gain of $10 and incorrect guesses were associated with monetary losses of $5. On each trial, participants were partnered with either the close friend who they put us in touch with, a stranger (confederate, said to be a past participant), or a non-human control (computer). To indicate partnership, from the beginning of the decision phase until the end of the outcome phase, an image appeared above the card displaying the partner with whom the outcome from each trial would be shared. Partners were presented in a block design that switched after 8 trials. Blocks were also grouped by outcome being either mostly reward or mostly loss (i.e., 6 being the condition of interest and 2 being neutral or of the opposite condition; cf. Barch et al., 2013). Participants were told that one trial would be picked at the end of the visit, and monetary outcomes would be split evenly with the partner on that given trial ($5 added to their final bonus payment for a win, and $2.50 removed for a loss). Money won with friends and strangers would be shared with them after, while money won with the computer would return to a pool of lab funds. At the end of the outcome phase, a 750 ms fixation cross would appear before the next decision phase. Participants completed two runs, each lasting 6 minutes and 54 seconds.

Immediately upon the fMRI scan’s completion, participants completed a follow-up portion of the task in which we administered post-session ratings of the emotional salience (e.g., “How did it feel…”) of winning or losing with each partner on a scale from −5 to 5 (e.g., “negative”, “neutral”, “positive”). Both the full task and follow-up were administered using PsychoPy 3 (Peirce et al., 2019). We note that two participants did not complete the partner rating portion post-scan due to time limitations at the end of the visit; thus, analyses that utilize this feature of the experiment include an N=43.

### Individual Difference Measures

#### Reward Sensitivity.

Reward sensitivity was defined by a composite score consisting of the sum of z-scores for the Behavioral Activation Scale (BAS; Carver & White, 1994) and the Sensitivity to Punishment/Sensitivity to Reward Questionnaire Reward subscale (SR; Torrubia & Tobeña, 1984). The BAS and SR subscales are reliable and valid measures of reward sensitivity (Alloy et al., 2006, 2012)

Both hypersensitivity and hyposensitivity to rewards have been linked to substance use (e.g., Alloy et al., 2009; Bart et al., 2021; Franken & Muris, 2006). To assess associations between brain response and both linear and nonlinear (i.e., aberrant) trait reward sensitivity, while avoiding overweighting the tails of the distribution (Büchel et al., 1998), we elected to normalize the values. To do so, we binned the composite reward sensitivity scores into deciles to produce an even distribution, then squared and mean-centered the scores to create an additional, quadratic measure that emphasizes aberrant reward sensitivity. Although we did not pre-register this strategy, this deviation allowed us to ensure detection of 2^nd^-order effects (i.e., U-shaped or inverted U-shaped responses) that are not driven by a single value.

#### Substance Use.

Substance use was operationalized as a composite score consisting of the sum of z-scores for the Alcohol Use Disorders Identification Test (AUDIT; Saunders et al., 1993) and the Drug Use Disorders Identification Test (DUDIT; Berman et al., 2002). The AUDIT is a 10-item self-report measure that assesses amount of alcohol consumption and frequency of alcohol-related problems. The DUDIT is an 11-item self-report measure that assesses frequency and disruptiveness of non-alcoholic drug use, containing references to a wide array of substances, including marijuana, amphetamines, and others. We used a sum of z-scores for AUDIT and DUDIT because our hypotheses related to all forms of substance use. Substance use scores also were binned into deciles to create an even distribution.

Given that we screened participants to exclude those who could not abstain from substance use, we consider our sample sub-clinical. Both scales have a threshold for clinically significant abuse. AUDIT scores of 1 to 7 are considered low-risk consumption, while scores of 8 to 14 are considered hazardous or harmful, and scores of 15 or more are classified as dependence. AUDIT scores in our sample range from 0 to 14 (mean=3.6, SD=3.5). DUDIT scores above 24 are indicative of dependence, while scores >1 for women or >5 for men are a large deviation from the mean (Basedow et al., 2021). DUDIT scores in our sample range from 0 to 28 (mean=2.3, SD=5.8). While two participants in the sample reached the dependence threshold for DUDIT (scores = 26, 28), their AUDIT scores were below threshold, and we consider our sample to be sub-clinical. Of the 45 participants included in the analyses, eight abstained from alcohol use. While AUDIT measures alcohol, DUDIT measures a wide variety of drug types. Within our sample, participants reported recreationally using Ritalin/amphetamine (n=14), marijuana (n=2), cocaine (n=1), and solvents/inhalants (n=2) within the year prior to their participation. The cocaine and solvent/inhalant users also used Ritalin/amphetamines. The remaining 29 participants did not use substances aside from alcohol, and a total of 21 did not partake in any form of substance use.

#### Self-Reported Social Closeness.

As part of the post-scan surveys, we assessed the degree of closeness between the MRI participant and their close friend with the ‘Inclusion of Other in Self’ (IOS; Aron et al., 1992) scale. While not initially part of the procedure, we followed up with participants to gather self-reports of IOS with the stranger (confederate) and a computer (non-human control) with whom they were partnered during the task. Follow-ups occurred after fMRI data collection was complete, between 1 month and 2.1 years after the initial appointment (mean = 247.2 days, median = 134.5 days). The IOS scores were assessed by comparing differences in closeness rating between each partner. No relationship was found between time elapsed after the initial appointment and differences in closeness rating (follow-up delay vs [friend - stranger] closeness: r = 0.19, p = 0.33; follow-up delay vs [friend - computer] closeness: r = 0.28, p = 0.15; follow-up delay vs [stranger - computer] closeness: r = 0.07; p = 0.72). Due to the follow-up nature of this data collection, there was participant attrition and we ultimately collected responses on the IOS for all three partners from N=28 participants.

### Behavioral Analyses

Behavioral measures were assessed in accordance with our pre-registration. We used a one-way repeated measures ANOVA to assess IOS scores across partners (friend, stranger, computer), and a 2×3 repeated measures ANOVA to assess ratings of emotional salience for wins and losses with each partner. Additionally, in analyses involving trait differences between individuals, we utilized composite substance use scores and composite reward sensitivity scores, as well as squared reward sensitivity scores to further isolate aberrance towards either extreme. In multiple linear regressions of behavioral data, we included differences in ratings for wins between each partner, as well as trait measures of substance use, reward sensitivity, aberrant reward sensitivity, and interaction terms between substance use and each of the two methods of assessing reward sensitivity. While we initially anticipated the inclusion of IOS and its interaction with other terms in our multiple linear regression, due to the limited sample of this measure we chose to exclude it from the regression model.

### Neuroimaging Data Acquisition

Functional images were acquired using a 3.0 Tesla Siemens PRISMA MRI scanner and a 20-channel head coil. Bold Oxygenation Level-Dependent (BOLD) sensitive functional images were acquired using a simultaneous multislice (multi-band factor = 2) gradient echo-planar imaging (EPI) sequence (240 mm in FOV, TR = 1,750 ms, TE = 29 ms, voxel size of 3.0 × 3.0 × 3.0 mm^3^, flip angle = 74°, interleaved slice acquisition, with 52 axial slices). Each run included 237 functional volumes. We also collected single-band reference images with each functional run of multi-band data to improve motion correction and registration. To facilitate anatomical localization and co-registration of functional data, a high-resolution structural scan was acquired (sagittal plane) with a T1-weighted magnetization-prepared rapid acquisition gradient echo (MPRAGE) sequence (224 mm in FOV, TR = 2,400 ms, TE = 2.17 ms, voxel size of 1.0 × 1.0 × 1.0 mm^3^, flip angle 8°). In addition, we also collected a B0 fieldmap to unwarp and undistort functional images (TR: 645 ms; TE1: 4.92 ms; TE2: 7.38 ms; matrix 74×74; voxel size: 2.97×2.97×2.80 mm; 58 slices, with 15% gap; flip angle: 60°).

### Preprocessing of Neuroimaging Data

Neuroimaging data were converted to the Brain Imaging Data Structure (BIDS) using HeuDiConv version 0.9.0 (Halchenko et al., 2019). Results included in this manuscript come from preprocessing performed using fMRIPrep 20.2.3 (Esteban et al., 2019, 2018), which is based on Nipype 1.4.2 (K. Gorgolewski et al., 2011, 2018). The details described below are adapted from the fMRIPrep preprocessing details; extraneous details were omitted for clarity.

#### Anatomical data preprocessing.

The T1-weighted (T1w) image was corrected for intensity non-uniformity (INU) with Ǹ4BiasFieldCorrection`, distributed with ANTs 2.3.3, and used as T1w-reference throughout the workflow. The T1w-reference was then skull-stripped with a *Nipype* implementation of the àntsBrainExtraction.sh` workflow (from ANTs), using OASIS30ANTs as target template. Brain tissue segmentation of cerebrospinal fluid (CSF), white-matter (WM), and gray-matter (GM) was performed on the brain-extracted T1w using `fast` (FSL 5.0.9). Volume-based spatial normalization to one standard space (MNI152NLin2009cAsym) was performed through nonlinear registration with àntsRegistration` (ANTs 2.3.3), using brain-extracted versions of both T1w reference and the T1w template. The following template was selected for spatial normalization: *ICBM 152 Nonlinear Asymmetrical template version 2009c* (TemplateFlow ID: MNI152NLin2009cAsym)

#### Functional data preprocessing.

For each of the BOLD runs per participant, the following preprocessing steps were performed. First, a reference volume and its skull-stripped version were generated by aligning and averaging 1 single-band reference (SBRefs). A B0-nonuniformity map (or *fieldmap*) was estimated based on a phase-difference map calculated with a dual-echo GRE (gradient-recall echo) sequence, processed with a custom workflow of *SDCFlows* inspired by the èpidewarp.fsl` script (http://www.nmr.mgh.harvard.edu/~greve/fbirn/b0/epidewarp.fsl) and further improvements in HCP Pipelines. The *fieldmap* was then co-registered to the target EPI (echo-planar imaging) reference run and converted to a displacements field map (amenable to registration tools such as ANTs) with FSL’s `fuguè and other *SDCflows* tools. Based on the estimated susceptibility distortion, a corrected EPI (echo-planar imaging) reference was calculated for a more accurate co-registration with the anatomical reference. The BOLD reference was then co-registered to the T1w reference using `flirt` (FSL 5.0.9) with the boundary-based registration cost-function. Co-registration was configured with nine degrees of freedom to account for distortions remaining in the BOLD reference. Head-motion parameters with respect to the BOLD reference (transformation matrices, and six corresponding rotation and translation parameters) are estimated before any spatiotemporal filtering using `mcflirt`.

BOLD runs were slice-time corrected using `3dTshift` from AFNI 20160207. First, a reference volume and its skull-stripped version were generated using a custom methodology of *fMRIPrep*. The BOLD time-series (including slice-timing correction when applied) were resampled onto their original, native space by applying a single, composite transform to correct for head-motion and susceptibility distortions. These resampled BOLD time-series will be referred to as *preprocessed BOLD in original space*, or just *preprocessed BOLD*. The BOLD time-series were resampled into standard space, generating a *preprocessed BOLD run in MNI152NLin2009cAsym space*. First, a reference volume and its skull-stripped version were generated using a custom methodology of *fMRIPrep*. Several confounding time-series were calculated based on the *preprocessed BOLD,* notably including framewise displacement (FD).

Additionally, a set of physiological regressors were extracted to allow for component-based noise correction (*CompCor*). Principal components are estimated after high-pass filtering the *preprocessed BOLD* time-series (using a discrete cosine filter with 128s cut-off) for anatomical component correction (aCompCor). For aCompCor, three probabilistic masks (CSF, WM and combined CSF+WM) were generated in anatomical space. The implementation differs from that of Behzadi et al. in that instead of eroding the masks by 2 pixels on BOLD space, the aCompCor masks are subtracted from a mask of pixels that likely contain a volume fraction of GM. This mask is obtained by thresholding the corresponding partial volume map at 0.05, and it ensures components are not extracted from voxels containing a minimal fraction of GM. Finally, these masks are resampled into BOLD space and binarized by thresholding at 0.99 (as in the original implementation). Components are also calculated separately within the WM and CSF masks. For each CompCor decomposition, the *k* components with the largest singular values are retained, such that the retained components’ time series are sufficient to explain 50 percent of variance across the nuisance mask (CSF, WM, combined, or temporal). The remaining components are dropped from consideration. The head-motion estimates calculated in the correction step were also placed within the corresponding confounds file. All resamplings can be performed with *a single interpolation step* by composing all the pertinent transformations (i.e., head-motion transform matrices, susceptibility distortion correction when available, and co-registrations to anatomical and output spaces). Gridded (volumetric) resamplings were performed using àntsApplyTransforms` (ANTs), configured with Lanczos interpolation to minimize the smoothing effects of other kernels.

Many internal operations of *fMRIPrep* use *Nilearn* 0.6.2, mostly within the functional processing workflow. For more details of the pipeline, see the section corresponding to workflows in *fMRIPrep*’s documentation (https://fmriprep.readthedocs.io/en/latest/workflows.html).

Further, we applied spatial smoothing with a 5mm full-width at half-maximum (FWHM) Gaussian kernel using FMRI Expert Analysis Tool (FEAT) Version 6.00, part of FSL (FMRIB’s Software Library, www.fmrib.ox.ac.uk/fsl). Non-brain removal using BET (Smith, 2002) and grand mean intensity normalization of the entire 4D dataset by a single multiplicative factor were also applied.

### Neuroimaging Analyses

#### Individual Level Analyses.

Neuroimaging analyses used FSL version 6.0.4 (Jenkinson et al., 2012; S. M. Smith et al., 2004). We focused on two types of analyses (activation and connectivity) to investigate how individual differences in linear and quadratic reward sensitivity, as well as sub-clinical substance use, were associated with BOLD responses. Both used individual level general linear models with local autocorrelation (Woolrich et al., 2001).

We conducted analyses focused on both activation and effective connectivity. Our first level of processing evaluated two models. The first focused on the neural activation evoked during the outcome phase of the task in each individual run and contained 10 regressors accounting for possible outcomes. These included win, loss, and neutral outcomes with friends, strangers, and computers (9 regressors), as well as missed trials.

The second model at this same level of processing focused on task-dependent changes in regional neural connectivity, using a bilateral ventral striatum seed region (VS; Oxford-GSK-Imanova atlas; Tziortzi et al., 2011). To estimate the changes in connectivity between feedback types (e.g., reward vs. punishment), we extracted the average time-course from this seed and added it as a physiological regressor to assess generalized psychophysiological interaction (PPI; Friston et al., 1997; McLaren et al., 2012; O’Reilly et al., 2012) with the VS for each contrast. Prior meta-analyses have shown that PPI leads to consistent and specific patterns of task-dependent connectivity (Di & Biswal, 2017; D. V. Smith et al., 2016; D. V. Smith & Delgado, 2017).

Since we conducted two runs of our task, we then took the mean of each contrast from the output of both runs of the first level activation and connectivity models, respectively. Of the 45 participants, 42 had both runs of the task, with three participants having had one of the two runs discarded due to excessive head motion, as per our pre-registered criteria. The run-level output for these individuals was included in the group level analyses.

#### Group Level Activation Analysis.

Our group level activation model evaluated activation during either the reward (win) or punishment (loss) trials for each of the three partners (friend, stranger, and computer), as well as a variety of contrasts examining the difference between activation with friends versus strangers and computers in the following four conditions: reward, punishment, reward versus punishment, and overall. All models included the following regressors of interest: substance use, reward sensitivity, reward sensitivity squared, an interaction of reward sensitivity and substance use, and an interaction of reward sensitivity squared and substance use. They also included two regressors of no interest that controlled for average whole-brain temporal signal-to-noise ratio and mean framewise displacement (derived from MRIQC) in each participant.

For our ROI-based activation analyses, we extracted activation from the VS (Oxford-GSK-Imanova atlas; Tziortzi et al., 2011) region-of-interest (ROI) and conducted 2 × 3 repeated measures ANOVAs to assess potential differences across outcome (win, loss) and partner (friend, stranger, computer) conditions. For contrasts of interest, we subtracted beta estimates of activation from one condition (e.g., reward with stranger) from another (e.g., reward with friend). Greenhouse-Geisser sphericity correction was automatically applied to within-subjects omnibus output before assessing for significance. Likewise, Bonferroni correction was applied to any post-hoc t-tests run on ANOVA results.

We also conducted exploratory whole-brain analyses to investigate regions outside of the VS that may be implicated in substance use, reward, and social processes. Group-level analyses were conducted using Randomise (Winkler et al., 2014). Z (Gaussianised T) statistic images were thresholded using clusters determined by Z>3.1 and a (corrected) cluster significance threshold of P=0.05 (Worsley, 2001).

#### PPI Group Level Analyses.

We followed the same process for PPI analyses of activation, using a nearly identical model. We used additional pre-registered target ROIs for our PPI model to assess connectivity between the VS and posterior cingulate cortex (PCC; Oxford-GSK-Imanova atlas), the medial and ventromedial prefrontal cortex (mPFC and vmPFC; Bhanji et al., 2019), and posterior temporoparietal junction (pTPJ; Mars et al., 2012b). As with the activation analyses, we also conducted whole-brain exploratory connectivity analyses within each of our contrasts of interest, using a VS seed. These whole-brain analyses included regressors to assess for mediations by substance use, reward sensitivity, reward sensitivity squared, an interaction of reward sensitivity and substance use, and an interaction of reward sensitivity squared and substance use.

### Deviations

As noted in the Introduction, as well as the Participants section, due in part to the COVID-19 pandemic, we had several deviations from our pre-registered procedures and hypotheses. We initially planned to recruit 100 participants and limit the sample to college students between the ages of 18–22. We intended to collect both closeness ratings (IOS) for each partner and emotional salience ratings for each partner. However, due to procedural error, we failed to collect the IOS for computer and strangers during the experimental session and were only able to collect these ratings as follow-ups from 28 participants. The 17 participants who did not respond to the follow-up were excluded from analyses we pre-registered regarding IOS results.

We initially pre-registered hypotheses that assessed the interaction of substance use and closeness ratings (IOS), in order to assess effects independent of reward sensitivity. However, our sample had a limited range of substance use that did not allow us to robustly assess effects outside of a sub-clinical range. To control for effects of substance use, we still included our composite substance use score in models and reported significant results and interactions with other measures of interest. Although we are still assessing the same neural regions of interest, our results focus more heavily on the pre-registered whole-brain activation analysis, as well as the exploratory VS region-of-interest connectivity analysis, than on the substance use hypotheses. Additionally, whereas we had pre-registered hypotheses that included the terms reward sensitivity and substance use, we had not pre-registered how we would operationalize these terms. As detailed in the Individual Differences Measures, for both reward sensitivity and substance use we collected two surveys each and then combined and binned the surveys to ensure a normal distribution. We included sub-clinical substance use and reward sensitivity regressors in our statistical models as planned, and elected to also report results that demonstrate the interaction of the two.

## Results

### Aberrant Reward Sensitivity Moderates Preference for Sharing Wins with Friends.

Our pre-registration sought to test whether self-reported reward sensitivity and substance use would each independently moderate how positively individuals rated the sharing of rewards with friends relative to strangers.

**Figure 2.**
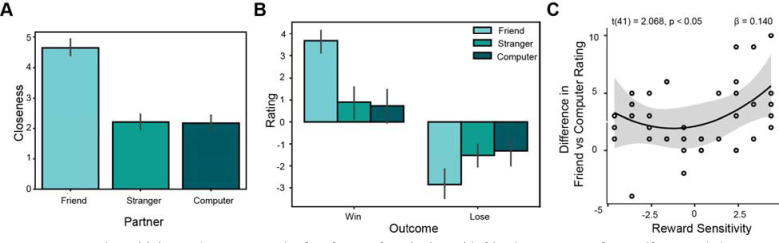
Reward sensitivity moderates strength of preference for winning with friends. (A) Means from self-reported closeness with each partner, collected via the Inclusion of Others (IOS) scale of 1–7. The IOS scale was missing from early data collection, and was recorded from some participants between 3 to 6 months after their visits. In total, N=28 completed the IOS. (B) Participants rated the emotional salience of winning and losing with each partner. An ANOVA demonstrated that ratings for winning and losing were significantly different across partners; the highest mean emotional salience in both conditions was with friends. (C) Participants in our sample who were either hyposensitive or hypersensitive to rewards (aberrant reward sensitivity) had a significantly greater boost in how they rated winning with a close friend, as opposed to a non-human partner, than those with more average reward sensitivity. However, reward sensitivity was not significantly associated with a difference in ratings of wins with friends as opposed to human strangers.

We first tested whether participants demonstrated different levels of closeness with each partner. Consistent with prior work from our group (Fareri et al., 2012, 2015), we found that average ratings for closeness with friends were significantly greater than with the confederate or computer partner (F(25, 2) = 30.496, p = 1.37e-09; fig. 2A). Mean closeness with friends was 4.64 out of 7 (SD = 1.42). Mean closeness with strangers was 2.21 (SD = 1.37). Mean closeness with the computer was 2.18 (SD = 1.42). We also conducted a 2×3 repeated measures ANOVA comparing self-report of emotional salience across partners and conditions. Wins with friends were significantly more emotionally salient than wins with strangers or computers (F(43, 2) = 35.277, p = 1.23e-11; fig. 2B). Mean rating for winning with friends was 3.65 (SD = 1.37). Mean rating for winning with strangers was 0.77 (SD = 2.42). Mean rating for winning with the computer was 0.53 (SD = 2.76). Mean rating for losing with friends was −2.86 (SD = 2.38). Mean rating for losing with strangers was −1.56 (SD = 1.85). Mean rating for losing with the computer was −1.47 (SD = 2.06). Together, these results suggest that participants’ subjective experiences in the task were shaped by our social manipulation.

We next ran a multiple linear regression of behavioral data assessing the effects of reward sensitivity, substance use, and differences in self-reported social closeness (e.g. [IOS rating for friend] - [IOS rating for stranger]) on the difference in ratings of emotional salience of winning with each partner (e.g. [win with friend] - [win with stranger]). As no significant effects emerged in this analysis, we ran a follow-up analysis to assess effects of reward sensitivity, reward sensitivity squared, substance use, the interaction of reward sensitivity and substance use, and the interaction of reward sensitivity squared and substance use, on the difference in emotional salience of winning with a close friend as opposed to a non-human partner (computer). While we found no relationship with sub-clinical substance use, we found that those with aberrant reward sensitivity (particularly high or low) were significantly more likely to prefer winning with friends over computers (t(41) = 2.381, p = 0.046; fig. 2C). This second order relationship with reward sensitivity extended to the difference between wins vs losses for friends as opposed to computers (t(41) = 2.341, p = 0.025). We followed up on this line of inquiry in our fMRI analyses, to determine whether a relationship with reward sensitivity is present in neural activation across partner types.

### Reward Sensitivity Moderates Enhancement of VS Activation in Response to Close Friends.

**Figure 3.**
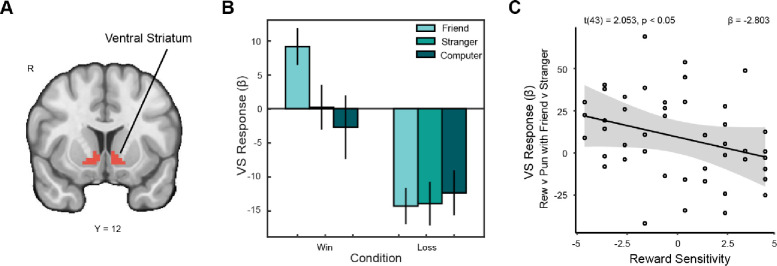
VS ROI response to reward with close friends moderated by reward sensitivity. (A) We focused on a pre-registered VS ROI (Tziortzi et al., 2011). (B) Within this region of interest, VS activation was significantly greater during wins with friends than wins with strangers. (C) We conducted a pre-registered analysis to determine whether reward sensitivity independently moderated VS activation for reward as opposed to punishment with friends as opposed to strangers. We found that as trait reward sensitivity increases, the overall difference in VS activation for outcomes shared with friends relative to strangers decreases. Those in our sample with low trait reward sensitivity showed a greater difference in VS enhancement when the context was a closer social relation than those with high reward sensitivity.

In our pre-registration we sought to investigate whether heightened trait reward sensitivity or sub-clinical substance use would be independently linked to increased ventral striatal responses to rewards shared with friends relative to strangers and computers. To obtain the most pronounced effect of social versus non-social activation, we assessed effects of social context by examining the difference between neural activation during the outcome phase of wins shared with friends vs those shared with computers (e.g., human vs non-human). We further distinguished effects of close social relations within social contexts by assessing the difference between neural response when sharing outcomes with friends vs with strangers (confederates). We first sought to replicate prior work (Fareri et al., 2012) showing that the VS reward response is enhanced in the context of close friends. To do so, we examined activation within the VS ROI (fig. 3A). Our two-factor repeated measures omnibus test assessing outcome across partners for the VS ROI demonstrated a significant interaction effect (F(1.56, 68.86) = 3.676, p = 4.10e-02; fig. 3B). A post-hoc pairwise t-test revealed that VS activation was significantly greater with a friend as the partner than with a stranger (t(44) = 2.53, p = 0.045). Although not a primary focus of the analyses, effects did not generalize to losses. This result provides further evidence for the enhancing effect of close social relations on VS activation during rewarding outcomes. We also hypothesized that the effect of sharing rewards with close friends on VS activation would be moderated by reward sensitivity and substance use, independent of one another. We did not find a relationship with substance use; however, the multiple linear regression model for neural responses revealed that within the activation extracted from the VS ROI for reward vs punishment in friends vs strangers, level of enhancement was significantly moderated by trait reward sensitivity (t(43) = −2.053, p = 0.047 ; fig. 3C). This finding indicates that those with higher trait reward sensitivity were more likely to demonstrate more similar VS activation when sharing outcomes with friends and strangers, whereas the enhancement from sharing with a close friend was more pronounced in those with low reward sensitivity.

We further examined differences in reward response between friends and strangers by running a whole-brain analysis. We found increased activation in a cluster extending into the ventral striatum (x, y, z: 8.5, −0.5, −3.5; ke = 53). This whole-brain result lends additional evidence to the importance of the VS in responding to context involving close social relations during receipt of reward.

Additionally, we assessed the relationship between substance use and difference in whole-brain reward response between friends and strangers. We identified a cluster in the superior temporal sulcus (STS; x, y, z: 54, −40, 11; ke = 37) whose activation when winning with friends vs computers was significantly moderated by level of sub-clinical substance use. As sub-clinical substance use levels increase, the difference in activation within this cluster of the superior temporal sulcus is significantly enhanced when sharing rewards with friends relative to strangers (t(43) = 3.783, p = 0.0005).

### Sharing Rewards with Close Friends Enhances Corticostriatal Connectivity.

Given the effects of sharing rewards with close friends vs strangers on activation, as well as the roles of trait reward sensitivity and self-reported sub-clinical substance use in moderating this relationship, we elected to assess how these conditions affect corticostriatal connectivity. We predicted that rewards shared with friends relative to strangers and computers would be associated with enhanced connectivity between the ventral striatum and several target regions. To investigate this hypothesis, we extracted activation in the target regions from our whole-brain psychophysiological activation model with the VS seed.

**Figure 4.**
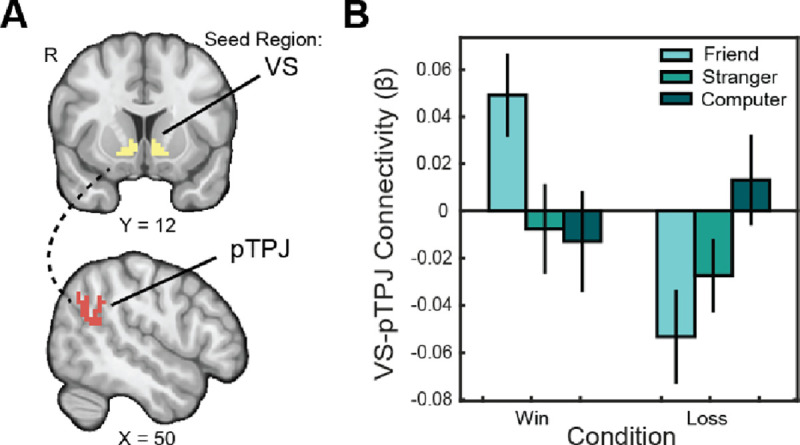
Social context enhances connectivity between VS-TPJ. (A) We pre-registered Regions of Interest (ROIs) in the VS (Tziortzi et al., 2011) and the posterior TPJ (Mars et al., 2012b). (B) We ran an analysis examining psychophysiological interactions between the VS seed and pTPJ target region to further probe for group differences in the effects of partner on reward processing. Connectivity between these regions for reward as opposed to punishment was significantly enhanced in the friend condition as opposed to the stranger condition.

A 2×3 repeated measures ANOVA revealed that connectivity between the VS and pTPJ was significantly enhanced in the context of friends relative to strangers or computers when comparing between outcomes (F(2, 88) = 7.841, p = 0.0007; fig. 4). A post-hoc pairwise t-test revealed a significantly greater reward response when sharing with a friend than with a stranger (t(44) = 2.59, p = 0.038) or with the computer (t(44) = −3.02, p = 0.013). This provides evidence that close social relations (close friend vs stranger), in addition to social context in general (close friend vs computer), enhance connectivity between the VS and pTPJ in response to reward. The ROI-ROI gPPI analysis did not find task-modulated connectivity between the striatum and the vmPFC, mPFC, or PCC.

We predicted that substance use would moderate connectivity between the ventral striatum seed and the pTPJ for rewards shared with friends as opposed to other partners, independently of reward sensitivity. To investigate this hypothesis, we ran our multiple linear regression model with the extracted VS-pTPJ connectivity for reward with friends vs strangers and friends vs computers. Sub-clinical substance use significantly moderated the difference in VS-pTPJ connectivity for rewards as opposed to punishments for friends as opposed to strangers (t(44) = 2.482, p = 0.017). Although we additionally predicted that this effect would be moderated by an interaction of IOS rating and self-reported substance use, due to the decreased sample size of participants who completed IOS, any results limited to these participants were underpowered. We did find, as predicted, that this effect was independent of individual differences in reward sensitivity.

**Figure 5.**
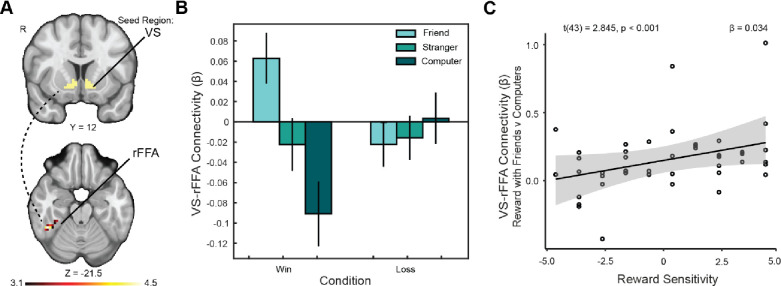
VS-rFFA connectivity is moderated by reward sensitivity. (A) We identified a cluster extending into the right temporo-occipital fusiform face area (rFFA) for which connectivity with the VS was significantly greater when sharing rewards with friends as opposed to computers (social context). The overlaying Z statistic image was generated using cluster-based thresholding with a (corrected) significance threshold of *P*=0.05. (B) There is a significant increase in connectivity between our VS seed and the rFFA when seeing a close friend’s face during receipt of reward as opposed to seeing a non-human partner. (C) Upon discovery of the rFFA cluster, in an exploratory analysis we tested whether connectivity was moderated by reward sensitivity independent of substance use. We found that the enhancement was moderated by trait reward sensitivity. Those in our sample with higher trait reward sensitivity saw a greater difference in VS-rFFA connectivity when sharing rewards with friends as opposed to computers.

To further assess the relationship between close social relations and corticostriatal connectivity, we ran a whole-brain PPI analysis with a VS seed. This analysis revealed that when sharing rewards with friends as opposed to a computer, connectivity between the VS and right fusiform area (rFFA; x, y, z: 44.5, −45.5, −21.5; ke = 29; fig. 5A) was enhanced (p<0.05). The result was bilateral, with an additional cluster from this contrast identified by the Harvard-Oxford Atlas as the lateral occipital cortex (x, y, z: −39.5, −81.5, −12.5; ke = 41). Interestingly, when comparing VS connectivity with the rFFA cluster for reward between strangers and computers, no significant enhancement was found. The enhancement from sharing with friends vs computers was significantly moderated by reward sensitivity (t(43) = 2.845, p = 0.007; fig. 5C). Reward sensitivity also moderated enhancement in the left cluster (t(43) = 2.670, p = 0.011). The result demonstrates that, within our sample, those with increased self-reported sensitivity to rewards showed a greater enhancement of connectivity between the VS-rFFA when seeing their friend’s face while winning. Differential VS-rFFA connectivity for reward with friends vs computers was also moderated by increased level of self-reported sub-clinical substance use (t(43) = 2.213, p = 0.033), independent of trait reward sensitivity.

Within the same set of whole-brain PPI analyses, we examined whether connectivity for rewards vs punishments shared with friends would be enhanced relative to both strangers and computers. Similar to our exploratory finding in the STS, we found connectivity between the VS and superior temporal gyrus (STG) within this contrast (x, y, z: 71.5, −33.5, 5.5; ke = 37). The enhancement (t(43) = −5.539, p = 5.22e-07) appeared when examining the moderating effect of the interaction between substance use and second order aberrant reward sensitivity. As reward sensitivity trends aberrant, higher levels of sub-clinical substance use were associated with an enhancement of VS connectivity with the STG when sharing rewards relative to punishments with friends as opposed to either of the other partners.

## Discussion

The current experiment investigated whether the social context in which rewards are experienced modulates neural activation and connectivity as a function of individual differences in reward sensitivity, independent from self-reported levels of substance use. Our results reveal that sharing rewards in the context of close friends enhances ventral striatal (VS) activation and corticostriatal connectivity with areas supporting social cognition, consistent with prior work from our group (Fareri et al., 2012). Importantly, our study expands upon this earlier work by demonstrating that reward sensitivity moderates the effects of varying social contexts on neural reward responses. Increased reward sensitivity is linked with more similar levels of neural response when sharing rewards with in-network and out-of-network peers, but a greater difference between playing with human partners and non-human entities. Increased levels of sub-clinical substance use also were linked to heightened neural activation and corticostriatal connectivity in social contexts. Our findings provide evidence that trait reward sensitivity and sub-clinical substance use independently moderate the effects of social context on neural reward response.

A rich body of literature has demonstrated evidence of corticostriatal response during reward-related tasks (J. P. Bhanji & Delgado, 2014; Dobryakova & Smith, 2022; Knutson et al., 2000; O’Doherty et al., 2017; Sazhin et al., 2020), noting links between the areas described as the “social brain” and the striatum when introducing social context (Fareri et al., 2022; Fareri & Delgado, 2014; Kwon et al., 2022; Lockwood et al., 2020). Likewise, trait reward sensitivity has been noted as a significant factor in neural reward response (Chat et al., 2022). Our results demonstrate that individuals with low reward sensitivity show greater enhancement of striatal activation when winning with friends, whereas those with high reward sensitivity show more similar activation for wins with either human partner. These results suggest that individuals with high reward sensitivity may be more susceptible to the effects of social context overall, irrespective of whether they are more close socially with the partner. We also found that sharing rewards with close friends enhances connectivity between the VS and the posterior temporoparietal junction (pTPJ). These two regions have previously been individually implicated in the enhancing effects of social context and close social relations (Fareri et al., 2022; Park et al., 2017). However, this result is the first to directly link connectivity between the VS and pTPJ with increased response to close friends relative to strangers, expanding upon evidence linking connectivity between the regions with pro-social sharing of monetary funds (Park et al., 2017). Furthermore, similar to past research into neural connectivity in social relative to non-social contexts (Haeger et al., 2014), we found that reward sensitivity is related to VS connectivity with the fusiform face area (FFA), indicating that individuals who are more sensitive to rewards may be particularly primed for reward activation when presented with a familiar face. Taken together, these results suggest that individuals with heightened reward sensitivity are increasingly susceptible to the effects of social context on VS activation and connectivity.

Similarly, we found that self-reported sub-clinical substance use levels moderated the degree to which social context influenced engagement of regions implicated in social cognition, such as the superior temporal sulcus (STS), as well as VS connectivity with the posterior temporoparietal junction (pTPJ) and right fusiform face area (rFFA). Previous work also has implicated these regions in the processing of social information (Bukowski, 2018; Deen et al., 2015; Genevsky et al., 2017; Mars et al., 2012a) as well as the processing of social alcohol cues (Groefsema et al., 2020; Maleki et al., 2022). Given the relevance of social context to substance use and abuse (Beard et al., 2022; Shadur & Hussong, 2014; Volkow et al., 2011), our results may indicate that increased activation in these regions is a common thread between increased sub-clinical substance use and responsivity to rewards experienced with peers. Such a relationship is consistent with the notion that peer presence can moderate the rewarding effects of drug use and predicts similarity in substance use habits among non-problematic users (Shadur & Hussong, 2014; Strickland & Smith, 2014). Although we hypothesized that level of self-reported substance use and aberrant trait reward sensitivity would be entirely independent, we found an interaction between the two in modulating connectivity between the VS and the superior temporal gyrus (STG) in social contexts. The STG considered a part of the “social brain” (Bigler et al., 2007), has shown differential volume and activation in individuals with alcohol use disorder (Brooks et al., 2014; Groefsema et al., 2020; Squeglia et al., 2011). Likewise, blunted reward response in the striatum has been linked to drug use levels and outcomes (Büchel et al., 2017; Luijten et al., 2017). Given that high or low reward sensitivity can determine the likelihood of increased substance use to remain sub-clinical or result in substance use disorders (Bart et al., 2021; Franken & Muris, 2006; Joyner et al., 2019; Nusslock & Alloy, 2017; Volkow et al., 2010), future research should investigate whether lower VS-STG connectivity in social contexts may be linked to risk for future substance use disorders in those with increased sub-clinical substance use.

We note that our work is accompanied by several limitations. We did not collect ratings of perceived closeness with each partner in the study from our full sample due to experimenter error, and thus, were underpowered for replicating prior effects relating to differences in closeness between friends and strangers (Fareri et al., 2012). Although the Inclusion of Other in Self scale (Aron et al., 1992) is a useful method of assessing perceived degree of social closeness, future studies should broaden their behavioral assessments with novel comparisons such as the similarity of autistic traits (Bolis et al., 2021) or neural response homophily (Parkinson et al., 2018) between friends. We also were unable to assess the pre-registered interaction between closeness with friends and substance use as a covariate in our analyses of neural response. Such behavioral data could provide a crucial link to answer whether perceived support modulates the moderating effects of substance use on the differential activation and connectivity within socially implicated regions. Future studies could include this covariate in larger sample sizes. Given that we limited the variability in substance use, ensuring that participants were below clinical cutoffs in order to control more precisely for the effects of reward sensitivity (Joyner et al., 2019), future work should assess the effect of clinically significant levels of substance use on processing rewards in social contexts. Additionally, our study had limited variability in participant age, focusing on young adults between the ages of 19–25. Given that adolescents and older adults show differential VS responses in social contexts (Fareri et al., 2022; Gadassi Polack et al., 2023; O’Brien et al., 2011; A. R. Smith et al., 2015; Telzer et al., 2013), the effects of reward sensitivity on monetary reward response in social contexts require further research across the human lifespan.

Despite these limitations, our findings provide important insights into the associations between individual differences in reward sensitivity, substance use, and the modulation of VS connectivity in response to rewards within social contexts. Our findings demonstrate novel links between reward sensitivity and sub-clinical substance use with the effects of social context in modulating corticostriatal connectivity, particularly underscoring the significance of close social relations in shaping neural responses to reward. Given links between aberrant reward sensitivity, social cognition, and suicidal ideation (Alloy et al., 2016; Nusslock et al., 2012; Senna et al., 2022; Szanto et al., 2012), as well as existing links between neural reward response and depression (Gotlib et al., 2010; Nelson et al., 2016), further research is warranted into whether individuals with mood disorders would show differential corticostriatal connectivity in social contexts based on their sensitivity to reward. Future longitudinal research should continue to investigate the predictive value of corticostriatal connectivity in clinical populations and explore interventions that modulate reward responses (Nagy et al., 2020), particularly in conjunction with social skill and support interventions (Ait Oumeziane et al., 2019; Narr et al., 2019; Piccirillo et al., 2021; Sequeira et al., 2021; Zhang et al., 2014) with the aim of preventing and addressing substance use and mood disorders. By demonstrating the modulatory effects of close social relations and their interplay with trait reward sensitivity and sub-clinical substance use, our findings pave the way for future research and interventions aimed at improving social support for individuals with aberrant reward sensitivity.

## Figures and Tables

**Figure 1.**
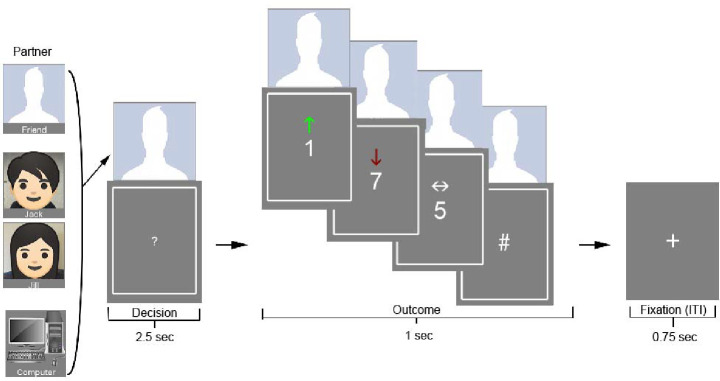
Task structure. Participants played a card guessing task (Delgado et al., 2000; Fareri et al., 2012) which partnered them with a computer, a gender-matched confederate (Jack/Jill), or a close friend. Players guessed whether the number on a card would be above or below 5, with choices between 1 and 9. A picture of the partner’s face remained above the card from the onset of decision-making through the end of the outcome phase. A green up-arrow indicated a correct guess and a monetary gain of $10.00; a red down-arrow indicated an incorrect guess and a monetary loss of $5.00; a white arrow pointing side-to-side indicated a neutral outcome with no money won or lost. Participants were informed prior to playing that the outcome of each round would be shared equally with the partner on the screen.

## Data Availability

Analysis code related to this project can be found on GitHub (https://github.com/DVS-Lab/istart-sharedreward). In addition, all data is available on OpenNeuro (https://identifiers.org/neurovault.collection:15006).
